# Defining Mild Stroke: Outcomes Analysis of Treated and Untreated Mild Stroke Patients

**DOI:** 10.1016/j.jstrokecerebrovasdis.2015.01.037

**Published:** 2015-04-20

**Authors:** Ilana Spokoyny, Rema Raman, Karin Ernstrom, Pooja Khatri, Dawn M. Meyer, Thomas M. Hemmen, Brett C. Meyer

**Affiliations:** 1 University of California, San Diego; 2 University of Cincinatti, Ohio

## Abstract

**Introduction:**

Mild deficit is a relative contraindication to administration of IV rtPA for acute ischemic stroke. However, what constitutes “mild” deficit is vague. Prior studies showed patients with mild strokes have substantial disability rates at hospital discharge and at 90 days. We investigated whether the application of a new definition altered the rates of disability overall, and assessed the effects of thrombolysis.

**Methods:**

This analysis included all adult acute ischemic stroke patients from a prospective registry of consecutive patients (UCSD SPOTRIAS database, 2003-2014) with 90-day mRS score available who were defined as “mild” using either: NIHSS 0-5 or a TREAT Task Force definition (NIHSS 0-5 and non-disabling based on pre-specified syndromes). Dichotomized 90-day mRS were compared between treated and untreated patients using the two definitions.

**Results:**

Of 802 ischemic stroke patients with mRS scores available, 184 had baseline mRS(0) and met TREAT criteria; 45(24.5%) were rtPA-treated. Among treated patients, 35.6% had 90-day mRS(2-6), versus 28.8% in the untreated group, a non-significant difference after adjusting for baseline NIHSS (p=0.47). None of the 45 treated patients had symptomatic hemorrhage. Outcomes were similar using the simpler NIHSS 0-5 definition.

**Conclusions:**

About one-third of mild stroke patients were not functionally independent at 90 days, irrespective of treatment or mild definition applied, calling into question the treatment efficacy of IV rtPA for mild strokes as well as what constitutes an appropriate definition of “mild”. Randomized studies are necessary to determine rtPA treatment efficacy in mild stroke patients.

## Introduction

Even almost 20 years after the initial positive trials, rtPA continues to be the only FDA approved therapy for acute stroke. The proportion of patients who receive thrombolysis is staggeringly low nationwide.^1^ As written, the exclusion criteria for the administration of recombinant tissue plasminogen activator (rtPA) limit the number of patients who are eligible to receive this therapy. Over time, and with more comfort in giving rtPA, the necessity of particular exclusion criteria has been called into question^2, 3^ with some studies, for example, focusing on the excluded combination of diabetes and prior stroke,^4^ and others on the age limit of 80 years within the 3 to 4.5 hour window.^5, 6^ More recently, focus has been on more accurately defining the intended meaning of the “rapidly improving stroke symptoms” exclusion criteria.^7^

The majority of patients with acute ischemic stroke are not treated with rtPA because they do no not reach the hospital soon enough. Of those patients who do present within the appropriate time window, 30-40% are excluded because of mild or rapidly improving stroke symptoms.^8-11^

The National Institute of Neurological Disorders and Stroke trials^12^ list “mild or rapidly improving symptoms” as a contraindication to treatment. The NINDS trials excluded a substantial number of strokes with minor presentations, and no patients were enrolled into that trial with isolated motor symptoms, isolated facial droop, isolated ataxia, isolated dysarthria, isolated sensory symptoms, or whose presentation was not captured by the NIHSS score (NIHSS=0).^13^ Other mild stroke cases were enrolled into the original trial in small numbers, but there remains lack of clarity regarding treatment of patients with these mild symptoms or even isolated symptoms.

The specific definition of “mild” has not been agreed upon universally, and there is variability in the interpretation and implementation among centers and even individual treating practitioners. One study defined “mild” as NIHSS 0-5, and found rates of rtPA administration to be 9% and symptomatic intracranial hemorrhage (sICH) of 1.9% among rtPA-treated patients.^11^ Using a definition of NIHSS 0-3, another study^14^ found significant variation (2.7-18.0%) in the frequency of mild strokes treated across different comprehensive stroke centers, and sICH rates of 1.2% among rtPA-treated patients.

In considering the definition of rapidly improving stroke symptoms (RISS), the TREAT Task Force investigators proposed a preliminary definition of disabling stroke as a combination of specific NIHSS scores and clinical criteria regarding the disabling nature of the deficits. We used the inverse of these criteria to define mild stroke (Table 1). We compared safety and functional outcomes among treated and untreated mild stroke patients to test the hypothesis that having a “mild” stroke is associated with good functional outcome, as well as to assess the benefit of thrombolysis in these patients. We used two definitions of mild stroke: a simple NIHSS cutoff (0-5) as well as the TREAT definition, to assess whether a more specific definition and thus group selection made a difference in outcomes or benefit of thrombolysis.

## Methods

This analysis included review of all adult acute ischemic stroke patients from a prospective registry of consecutive stroke code patients (the UCSD SPOTRIAS database, 2003-2014) with 90 day mRS score available who could be classified as “mild” by various definitions. Stroke codes are routinely called on patients presenting within 12 hours of onset of neurologic deficit. Patients treated with IV rtPA and untreated patients were included; patients treated with mechanical or other agent were excluded. Patients were grouped according to baseline modified Rankin Score (mRS) and definition of “mild” (Table 2).

Two definitions of “mild” were used. The first analysis was performed with mild stroke being defined as NIHSS 0-5, regardless of specific symptoms. The second analysis employed a recently proposed definition derived from the TREAT Task Force. The TREAT Task Force proposed a set of criteria in which any of the following would classify someone as having a disabling stroke: complete hemianopsia (≥2 on NIHSS question 3), severe aphasia (≥2 on NIHSS question 9), visual or sensory extinction (≥1 on NIHSS question 11), any weakness limiting sustained effort against gravity (≥2 on NIHSS question 5 or 6), any deficits that led to a total NIHSS >5, or any remaining deficit considered potentially disabling in the view of the patient and the treating practitioner. Using the inverse of this definition of disabling symptoms, the TREAT-derived definition of mild stroke was created (Table 1).

Group 1 consisted of patients with baseline mRS(0) and mild defined as NIHSS 0-5. Group 2 consisted of patients with baseline mRS(0) and the TREAT-derived mild definition. These two analyses were performed with baseline mRS score of zero only, in order to better isolate the symptoms of a mild stroke.

Group 3 consisted of patients with any baseline mRS(0-5) and mild defined as NIHSS 0-5. Group 4 consisted of patients with any baseline mRS(0-5) and the TREAT-derived mild definition. These analyses were performed to limit the selection bias which may be present in analyses 1 and 2, which included only previously normal patients. In addition, these analyses, by including all baseline mRS scores, allowed for greater patient numbers.

The patients who were determined subjectively by the clinician at the time of treatment decision to be “too mild to treat” (TMT) were identified within each analysis and subgroup analyses were performed. This served as surrogate for the “clinical judgment” portion of the definition proposed by the TREAT task force.

Based on these analysis groups, baseline characteristics of gender, race, ethnicity, hypertension, diabetes, atrial fibrillation, prior stroke, smoking status, age, baseline NIHSS, and baseline glucose were assessed for both the rtPA treated and untreated “mild” stroke patients. Dichotomized 90-day mRS scores (0-1 vs. 2-6) and frequency of presentation with each NIHSS criteria were compared between treated and untreated patients, as well as the TMT group. Rates of symptomatic intracerebral hemorrhage (sICH) were also reported.

Since this is an exploratory study, results are reported as estimates with the 95% confidence intervals without any adjustment for multiple comparisons. Statistical analyses was performed using the software R (version 3.0.2, http://www.r-project.org).

## Results

There were 802 acute ischemic stroke patients included in our database who had 90 day mRS scores available. These patients did not represent mimics or TIAs; after analysis of the clinical course and imaging, multiple stroke practitioners determined them to be ischemic strokes. This consensus was reached at a weekly stroke team meeting during which all aspects of the case were presented. When analysis was limited to baseline mRS of zero (analyses 1 and 2), we identified 276 (34.4%) patients with NIHSS 0-5 and 184 (22.9%) patients met criteria for the TREAT-derived definition of mild stroke. Including all baseline mRS scores (analyses 3 and 4), 374 (46.7%) patients had NIHSS 0-5 and were either treated with r-tpA or untreated (patients treated with mechanical or other agent were excluded); 251 (32.3%) patients met criteria for the TREAT-derived definition of mild stroke. We treated between 23 to 30 percent of mild strokes. This number varied depending on the baseline mRS included and definition of mild stroke that was used. Treated patients consistently had higher baseline NIHSS scores. Treated patients were more likely to get NIHSS points for arm weakness and aphasia in every analysis, with other questions showing significance in certain populations. Among patients with baseline mRS of zero, there were no cases of sICH. In the groups which included all baseline mRS, sICH rate was 2.9 to 4 percent. Between 57 and 64 percent of mild patients were excluded from treatment due to being subjectively too mild to treat (TMT). TMT patients had poor outcomes 25 to 30 percent of the time. There was a significant proportion of patients in each analysis whose *only* exclusion criteria was too mild to treat (TMT-only). These patients had poor outcomes 15 to 30 percent of the time.

## Group 1: mRS=0, mild defined as NIHSS=0-5

Of the 276 Group 1 patients, 83 (30%) were rtPA treated. Treated and untreated patients had similar baseline characteristics except that the treated group had higher baseline NIHSS (mean 3.45 vs. 2.0, p<0.0001). In the treated group, 37.4% had mRS 2-6 at 90 days, compared to 31.1% in the untreated group, a non-significant difference (p=0.44) adjusting for baseline NIHSS. None of the 83 rtPA treated Group 1 patients had sICH. The treated patients were more likely to get NIHSS points for LOCQ (p=0.005), motor left arm (p=0.007), motor right arm (p=0.005), and aphasia (p<0.0001).

Of the 193 untreated Group 1 patients, 113 (58.5%) were reported by the clinician to be excluded from rtPA because the deficit was “too mild to treat” (TMT). The next most common exclusion criteria (after TMT) were presentation over 3 hours (47.7%) and rapidly improving symptoms (23.3%). The 113 TMT patients had similar baseline characteristics to the rest of the untreated population except for lower baseline NIHSS (mean 1.65 vs 2.61, p<0.0001) and baseline glucose (mean 129 vs. 147, p=0.0002). The TMT patients were less likely to get NIHSS points for LOCQ (p=0.016) and aphasia (p=0.002) than the rest of the untreated patients. Among the TMT group, 29.2% had mRS 2-6 at 90 days, compared with 33.8% of other untreated mild patients, a non-significant difference (p=0.48) adjusting for baseline NIHSS and glucose.

Of 113 TMT, 40 had no other rtPA exclusion criteria (including time). These TMT-only patients had similar baseline characteristics to treated patients except lower baseline NIHSS and glucose, and had non-significant difference in outcomes (25% vs 37.35%, p=0.67). TMT-only were less likely to get NIHSS points for motor left arm (0.016), motor right arm (0.0339), or aphasia (<0.0001).

## Group 2: mRS=0, TREAT-derived mild definition

Of the 184 Group 2 patients, 45(24.5%) were rtPA treated. Treated and untreated patients had similar baseline characteristics except that the treated group had higher baseline NIHSS (mean 3.29 vs. 1.81, p<0.0001). In the treated group, 35.6% had mRS 2-6 at 90 days, compared to 28.8% in the untreated group, a non-significant difference (p=0.47). None of the 45 treated patients had sICH. The treated patients were more likely to get NIHSS points for gaze (p=0.041), facial palsy (p=0.018), motor left arm (0.002), motor right arm (p=0.002), motor left leg (0.014), and aphasia (0.004).

Of the 139 untreated Group 2 patients, 87 (62.6%) were TMT. The next most common exclusion criteria (after TMT) were over 3 hours (46.8%) and rapidly improving symptoms (23.7%). The 87 TMT patients had similar baseline characteristics to the rest of the untreated population except for lower baseline NIHSS (mean 1.49 vs 2.33, p=0.0013) and baseline glucose (mean 124 vs. 147, p<0.0001). The TMT patients were less likely to get NIHSS points for LOCQ (p=0.016) and aphasia (p=0.008) than the rest of the untreated patients. Among the TMT patients, 25.3% had mRS 2-6 at 90 days, compared with 34.6% of other untreated mild patients, a non-significant difference (adjusted p=0.95).

Of 87 TMT, 33 had no other rtPA exclusion criteria (including time). These TMT-only patients had similar baseline characteristics to treated patients except lower baseline NIHSS and glucose. In this subgroup, 15.2% had mRS 2-6 at 90 days, compared with 35.6% of treated patients in this analysis, a nonsignificant difference (adjusted p=0.28). TMT-only were less likely to get NIHSS points for facial palsy (0.02), motor left arm (0.003), motor right arm (0.020), motor left leg (0.0339), or aphasia (0.0004).

Groups 3 and 4 (using baseline mRS 0-5) had similar results (Table 3).

## Discussion

Using both definitions of mild stroke (either simply NIHSS 0-5 or the TREAT-derived definition) we were unable to convincingly show a difference in outcomes in treated versus untreated mild stroke patients. Additionally, we did not show a difference in outcomes based on the definition used. Regardless of treatment being given, or which definition was used, 25 to 30 percent of patients did not have good functional outcome at 90 days. This emphasizes the current equipoise for efficacy of IV rtPA for mild strokes.

The variability of outcomes of mild strokes, with or without thrombolysis, has been surprising. Multiple studies have shown that about a third of untreated mild patients were left either dependent or dead at 90 days, bringing into question the initial decision not to treat.^8-10, 15, 16^ In an analysis of the “Get With The Guidelines” dataset,^9^ 31% of patients presenting within 2 hours did not receive rtPA solely because of mild/improving symptoms, and 28% of 29200 patients with mild/improving stroke not treated with rtPA could not be discharged home.

Our treatment rate of mild stroke patients was high, at 23 to 30 percent. This is at the top end of rates published.^11, 14^ This is likely attributable to our aggressive treatment approach and not using a low NIHSS cutoff as an absolute treatment exclusion criteria. Despite an aggressive approach, between 57 and 64 percent of mild patients were excluded from treatment due to being subjectively too mild to treat (TMT).

Treated patients consistently had higher baseline NIHSS scores, and it is possible that the treated group would have had a worse outcome had they not received treatment. This may indicate that an even lower NIHSS cutoff may be appropriate to incorporate into a definition for mild stroke which will correlate with outcomes. A definitive conclusion cannot be made due to the study design and difference in baseline NIHSS between treated and untreated mild stroke patients. Treated mild stroke patients were more likely to get NIHSS points for arm weakness and aphasia in every analysis, with other questions showing significance in certain populations. There have not been prospective studies determining whether presence of specific NIHSS criteria influence outcome of mild stroke patients. One retrospective study addressing this issue concluded that the individual components of the baseline NIHSS were not independent predictors of long-term prognosis for patients with mild stroke. The authors of that study argue against the practice of withholding reperfusion treatment in patients with mild stroke when the types of baseline NIHSS deficits are perceived to be non-disabling.^17^

Among patients with baseline mRS of zero, there were no cases of sICH. In the analyses which included all baseline mRS, sICH rates were 2.9 to 4 percent. These sICH rates are on par with published rates.^11, 14^

The analyses were performed for both baseline mRS of zero (analyses 1 and 2) and all baseline mRS (analyses 3 and 4). The reasoning behind limiting the first two analyses to mRS of zero is the difficulty of identifying mild symptoms in a patient with significant neurologic impairment at baseline. A patient with significant baseline deficits may have a baseline elevated NIHSS score which when combined with mild deficits, excludes them from the analysis due to total NIHSS score.

TMT patients had poor outcomes 25 to 30 percent of the time. TMT patients were subjectively determined to be too mild to treat, and primarily this determination was made by the physician's interpretation of level of disability incurred by the stroke. This degree of poor outcomes is remarkable and indicates practitioner inability to correctly predict ultimate severity of mild stroke symptoms.

It has been argued that thrombolysis should not be withheld in the case of mild stroke symptoms, because many of these patients will have poorer than expected outcomes, and because there is likely a low risk of hemorrhagic complications in these patients.^18^ Our study results corroborate that many of these patients have poorer than expected outcomes, but we did not show a clear benefit to thrombolysis.

Although there have been many assumptions regarding the outcomes of mild stroke patients, the best definition and true outcomes for this population are not well known. This lack of definition has provided the impetus for this study, but also contributed to one of its limitations. The TREAT-derived definition was used as it appeared to be more specific and the goal was to isolate truly “mild” patients. However, there was no clear difference in outcomes prediction when using the TREAT definition rather than a simple NIHSS cutoff. Without a consensus on the extent of stroke, or particular symptoms, necessary to clinically determine that a stroke is disabling, there will be significant variation in the patients excluded due to their symptoms being “too mild”. Given the duration of this observational study, many observers were involved in scoring the NIHSS and 90-day mRS which may have resulted in variability in scoring. In addition, whether a symptom is “disabling” hinges heavily on the patient's baseline functional status and activity level.

The hesitation of many providers to treat mild stroke patients with rtPA is based on their perception of the risk/benefit ratio of treatment. Giving a treatment with a historical 6.4% symptomatic hemorrhage in the treatment arm of the landmark study^12^ seems risky when the assumed outcome of the patient in question is spontaneous recovery. It is notable that mild strokes were largely excluded from the NINDS trials^13^, and that many studies^19-21^ have since demonstrated safety of treatment of TIAs and stroke mimics with rtPA. Our data shows a likely lower risk of ICH in our treated mild cohort, but only a prospective study comparing treated versus non-treated patients can confirm these results.

Our study augments the literature proposing and assessing a new definition of mild stroke, and showing that neither the definition used nor the treatment given made a difference in the poorer than expected outcomes of mild stroke patients. This study was likely underpowered to detect a significant treatment effect and is limited by its design. In addition, the poor outcomes of mild stroke patients were not necessarily caused by the stroke with which they presented. There is the possibility that the poor 90-day outcome was a result of patients having another stroke, hemorrhaging into the current stroke, or another non-neurologic cause of disability in the interim. There remains equipoise regarding best acute treatment strategies for mild stroke patients. Randomized studies are necessary to determine rtPA treatment efficacy in mild stroke patients.

## Figures and Tables

**Table 1:** TREAT-derived Mild Stroke Criteria

NIHSS 0-5 AND absence of any of the following: - complete hemianopsia (≥2 on NIHSS question 3) - severe aphasia (≥2 on NIHSS question 9) - visual or sensory extinction (≥1 on NIHSS question 11) - any weakness limiting sustained effort against gravity (≥2 on NIHSS question 5 or 6) - any remaining deficit considered potentially disabling in the view of the patient and the treating practitioner

**Table 2:** Study Groups

	Definition of Mild Stroke	
	NIHSS 0-5 (N)	TREAT-derived mild definition (N)
Baseline mRS 0	Group 1 (276)	Group 2 (184)
Baseline mRS 0-5	Group 3 (374)	Group 4 (251)

**Table 3:** Outcomes of Study Groups

	Group 1	Group 2	Group 3	Group 4
Mild Definition	NIHSS 0-5	TREAT	NIHSS 0-5	TREAT
Baseline mRS	0	0	0-5	0-5
Number of Patients	276	184	374	251
treated	83 (30%)	45 (24.5%)	105 (28.1%)	58 (23.1%)
untreated	193 (70%)	139 (75.5%)	269 (71.9%)	193 (76.9%)
TMT (too mild to treat)	113	87	155	123
TMT-only	40	33	57	46
Poor outcome at 90 days				
treated vs untreated	37.4% vs 31.1%, adjusted p=0.44	35.6% vs 28.8%, adjusted p=0.47	38.1% vs 32.3%, adjusted p=0.67	32.8% vs 29.0%, adjusted p=0.47
TMT vs rest of untreated	29.2% vs 33.8%, adjusted p=0.48	25.3% vs 34.6%, adjusted p=0.95	29.7% vs 36.0%, adjusted p=0.95	26.0% vs 34.3%,adjusted p=0.59
TMT-only vs treated	25% vs 37.35%, adjusted p=0.67	15.2% vs 35.6%, adjusted p=0.28	29.8% vs 38.1%, adjusted p=0.72	21.7% vs 32.8%, adjusted p=0.96
Symptomatic ICH (among treated)	0	0	3 (2.9%)	2 (4.0%)
